# The Student’s Health Creed

**Published:** 1913-05

**Authors:** 


### The Student's Health Creed.

I believe my body and good health are sacred. If I am sick, it will very probably be because I have violated some one or more of nature's laws of health.

I will study nature's laws of health and will obey them for my own sake.

I will not suck my fingers, or pick my nose, or wipe my nose on my hand or sleeve, for these practices are unsanitary and very impolite.

I will not wet my fingers in my mouth when turning the leaves of books.

I will not put pencils in my mouth or wet them with my lips.

I will not put pins or money in my mouth.

I will not buy or use chewing gum nor buy and eat cheap candies.

I will only use my mouth for eating good, plain food, drinking pure water and milk, and for saying good and kind words.

I will always chew my food thoroughly, and never drink whisky or wine.

I will strive against the habit of "clearing my throat," because it is nearly always unnecessary, and may be disagreeable to others.

I will not cough or sneeze without turning my face and holding a piece of paper or handkerchief before my mouth. Polite people never cough in public if they can prevent it.

I will keep my face, hands and finger nails as clean as possible.

I will not spit on the floor, stairways or sidewalks, and will try not to spit at all. Ladies and gentlemen do not spit.

I will wash my mouth every morning on getting up and at night in going to bed, and I will use a tooth brush if I can get one.

I will be clean in body, clean in mind, and avoid all habits that may give offense to others.

I will get all the fresh air I can and will open wide my bedroom windows when I go to bed.—*Indiana State Board of Health.*

